# Supplementary material for: Single Cell Transcriptomic and Chromatin Profiles Suggest Layer Vb Is the Only Layer With Shared Excitatory Cell Types in the Medial and Lateral Entorhinal Cortex
Source: Front Neural Circuits. 2022 Jan 26;15:806154. doi: 10.3389/fncir.2021.806154 (PMC8826650; doi:10.3389/fncir.2021.806154)
Supplement: Supplementary file 14 [file Data_Sheet_1.ZIP › Exc_vs_Inh_Motifs/homerResults/motif8.similar.html]

motif8

## Information for motif8

C
A
G
T
C
G
A
T
C
G
T
A
C
G
T
A
C
T
G
A
T
C
G
A
G
C
T
A
T
C
G
A
  
Reverse Opposite:  

A
G
C
T
C
G
A
T
A
G
C
T
G
A
C
T
A
C
G
T
C
G
A
T
G
C
T
A
G
T
C
A
  

|  |  |
| --- | --- |
| p-value: | 1e-291 |
| log p-value: | -6.708e+02 |
| Information Content per bp: | 1.801 |
| Number of Target Sequences with motif | 24254.0 |
| Percentage of Target Sequences with motif | 57.50% |
| Number of Background Sequences with motif | 5285.5 |
| Percentage of Background Sequences with motif | 48.62% |
| Average Position of motif in Targets | 337.0 +/- 246.4bp |
| Average Position of motif in Background | 316.6 +/- 269.4bp |
| Strand Bias (log2 ratio + to - strand density) | -0.0 |
| Multiplicity (# of sites on avg that occur together) | 1.82 |
| Motif File: | file (matrix) reverse opposite |

### Similar de novo motifs found

|  |  |  |  |  |  |  |  |
| --- | --- | --- | --- | --- | --- | --- | --- |
| Rank | Match Score | Redundant Motif | P-value | log P-value | % of Targets | % of Background | Motif file |
| 1 | 0.667 | A C G T A C G T C G T A A C G T C T G A A C G T C G T A C G T A | 1e-271 | -625.385595 | 9.59% | 5.33% | motif file (matrix) |
| 2 | 0.699 | C G A T A C G T A C G T A C G T A C T G A C G T A C G T C G T A | 1e-165 | -382.165511 | 76.97% | 71.03% | motif file (matrix) |
| 3 | 0.769 | A C G T C G T A C G T A C G T A C G T A C G T A C G T A A C G T C G T A A G T C | 1e-115 | -265.560327 | 67.48% | 62.14% | motif file (matrix) |
| 4 | 0.748 | C G T A A C G T A C G T A C G T A C G T A C G T C G T A C G T A C G T A C G T A | 1e-98 | -226.858475 | 69.96% | 65.12% | motif file (matrix) |
